# Efficacy and safety of traditional Chinese medicine treatment for idiopathic pulmonary fibrosis: An exploratory, randomized, double-blinded and placebo controlled trial

**DOI:** 10.3389/fphar.2022.1053356

**Published:** 2022-10-28

**Authors:** Jiansheng Li, Xue-qing Yu, Yang Xie, Shu-guang Yang, Limin Zhao, Miao Zhou, Yong Meng

**Affiliations:** ^1^ Collaborative Innovation Center for Chinese Medicine and Respiratory Diseases Co-constructed by Henan Province and Education Ministry of China, Henan University of Chinese Medicine, Zhengzhou, Henan, China; ^2^ Henan Key Laboratory of Chinese Medicine for Respiratory Disease, Henan University of Chinese Medicine, Zhengzhou, Henan, China; ^3^ Department of Respiratory Diseases, The First Affiliated Hospital of Henan University of Chinese Medicine, Zhengzhou, Henan, China; ^4^ Department of Respiratory Diseases, Henan Provincial People’s Hospital, Zhengzhou, Henan, China; ^5^ Department of Respiratory Diseases, The Third Affiliated Hospital of Henan University of Chinese Medicine, Zhengzhou, Henan, China; ^6^ Department of Respiratory Diseases, Henan Province Hospital of Traditional Chinese Medicine, Zhengzhou, Henan, China

**Keywords:** idiopathic pulmonary fibrosis, Chinese medicine, syndrome differentiation, efficacy, randomized controlled trial

## Abstract

**Background and objective:** Idiopathic pulmonary fibrosis (IPF) is a critical disease, with limited treatments available. Clinical practices show that traditional Chinese medicine (TCM) has certain efficacy. This study was preliminarily to evaluate the efficacy and safety of TCM treatment based on syndrome differentiation in IPF.

**Methods:** A study design of exploratory, multi-centers, randomized, double-blinded, placebo controlled trial has been adopted. A total of 80 IPF patients from four sub-centers were enrolled. All the patients were randomly assigned into TCM group (TCMG) or control group (CG) in 1:1. Patients in TCMG were given CM granules, as patients in CG given with the placebo of CM granule. All the patients received a 26-week treatment. The efficacy was assessed by acute exacerbations (AEs) of IPF, pulmonary function, clinical symptoms, dyspnea scores (mMRC), health-related quality of life (HRQoL), 6-min walk test (6MWT) and all-cause mortality. Safety has also been assessed.

**Results:** A total of 67 patients completed the trial with 35 in TCM group and 32 in control group. Meaningful differences have been observed in mean changes in AEs (−1.56 times; 95% CI, −2.69 to −0.43, *p* = 0.01), DLco% (5.29; 95% CI, 0.76 to 9.81, *p* = 0.02), cough scores (−0.38 points; 95% CI, −0.73 to −0.04, *p* = 0.03), and 6MWT (30.43 m; 95% CI, 2.85 to 58.00, *p* = 0.03), with no statistical differences in FEV1, FVC, expectoration, chest tightness, Shortness of breath, Fatigue, Cyanosis, mMRC, CAT, SF-36, and SGRQ total scores in 26 weeks after treatment than before treatment. At of the end of follow-up, a total of 10 patients died, including three and seven in the TCM and control group respectively. And the HR (Hazard ratio) for CM granules in all-cause mortality was 0.39 (95% CI, 0.10–1.52). The drug-related adverse events were not observed.

**Conclusion:** CM granules, as compared with placebo, could reduce frequencies of AEs, improve pulmonary function, HRQoL, exercise capacity and symptoms and signs for IPF to some extent with acceptable side-effect.

## 1 Introduction

Idiopathic pulmonary fibrosis (IPF) is a progressive and ultimately fatal interstitial lung disease (Raghu et al., 2011; Gao et al., 2021). With worsened dyspnea and an increasing loss of pulmonary function, IPF patients will have poor health-related quality of life (HRQoL) (Hopkins et al., 2016). It has also caused an increasing social-economic burden (Raimundo et al., 2016; Maher et al., 2021; Cox et al., 2022). Researches show that pirfenidone and nintedanib could be certain effective to IPF (Wu et al., 2021; Pitre et al., 2022), which were also recommended by the guideline (Raghu et al., 2015). However, its application has been limited by side effects and high prices. And the case fatality rate is still on the rise (Dove et al., 2019). It is urgent to develop other effective treatments and strategies to manage IPF.

TCM has a long history and definite efficacy on respiratory diseases. IPF can be treated referring to *fei-wei* or *fei-bi*. According to TCM theory that lung is the dominator of qi and kidney is the root of qi, they can both affect the development of IPF. So, the methods of regulating and reinforcing lung and kidney have been the most commonly used treatment in clinical practice for TCM. In previous studies, our research team also found that it can reduce the incidence of acute exacerbations (AEs) of IPF and delay the loss of lung function. However, most of the present studies are limited to summary of clinical experiences. It is urgent to manage further researches to obtain evidence-based supports. In our previous literature research, we concluded that the common TCM syndromes of stable IPF included lung-qi deficiency, lung-kidney qi deficiency, yin deficiency and internal heat, meanwhile had prescriptions for each syndromes of Bao-fei Hua-xian (Bu-fei Yi-qi) granule, Jin-shui Huan-xian (Bu-fei Yi-shen) granule, and Yang-qing Kang-xian (Yang-yin Qing-re) granule, respectively. We systematically searched the clinical trial registration platform and other related databases. This is the first registered RCT for TCM in treating IPF. So, we have performed this study to evaluate the efficacy and safety of CM granule based on above prescriptions for each syndrome of IPF. The results will also provide evidence-based supports for TCM treatment in IPF and critical references for further TCM studies. The study protocol has been registered in www.chictr.org.cn (ChiCTR-IIR-17013532) and published in *JIM* (Yu et al., 2020).

## 2 Subjects and methods

### 2.1 Study design

The study was an exploratory, randomized, double-blinded, placebo controlled, and parallel-group trial, performed in four sub-centers in Zhengzhou China, which include the First Affiliated Hospital of Henan University of CM, Henan Province Hospital of CM, Third Affiliated Hospital of Henan University of CM, and Henan Provincial People’s Hospital. An expert committee, included clinicians, statisticians and ethics experts, had been set up to perform the study design. The trial has been approved and supervised by Ethic Committee of the First Affiliated Hospital of Henan University of CM.

### 2.2 Patients

Patients were eligible to participate in the trial if they were aged 40–80 years old and met the diagnosis criteria of stable IPF (Raghu et al., 2011) 1 and syndrome differentiation of TCM (Lung qi deficiency syndrome, yin deficiency and internal heat Syndrome and of lung and kidney qi deficiency syndrome) (Professional Committee of Pulmonary Diseases of China Association of Chinese Medicine, 2012). They should not participate in any other trial within 1 month before enrollment. It was also necessary to sign informed consent before entering the study for all the subjects.

However, the patients with AEs of IPF, pregnancy or breast-feeding should be excluded, as should the subjects with obnubilation, dementia or mental disorders. Patients with severe cardiac insufficiency, severe liver and kidney diseases (ALT, AST, BUN, and Cr are more than twice the value of normal upper limit.) or being allergic to any of the used drugs should not be involved in the study.

Traditional Chinese medicine syndromes differentiation have been performed referring to the following diagnostic criteria:

Lung qi deficiency syndrome: ① Cough, or dyspnea, or shortness of breath; ② Fatigue with worsen symptom when moving; ③ Spontaneous sweating, which is also aggravated when moving; ④ Afraid of wind and cold, or easy to catch cold; ⑤ The tongue is light, or the pulse is thin or weak. On the basis of items ①, any other plus two items among ②, ③, ④, and ⑤ should be met.

Yin deficiency and internal heat syndrome: ① Dyspnea or shortness of breath; ② Dry cough, or cough with little or unpleasant sputum; ③ Dry mouth or throat; ④ Hands, feet and hearts are hot or hot in the afternoon; ⑤ Night sweat; ⑥ The tongue is red, or has little or no coating with dry or thin veins. On the basis of items ① and ②, any other plus two items among③, ④, ⑤, and ⑥ should be met.

Lung and kidney qi deficiency syndrome: ① Dyspnea, or cough, or shortness of breath, and symptoms will be worsen when moving; ② Mental weakness and fatigue or spontaneous sweating, with worse condition when moving; ③ Easy to catch a cold, or afraid of wind and cold; ④ Soreness of waist and knees; ⑤ Tinnitus or dizziness; ⑥ The face is puffy; ⑦ Frequent urination, increased nocturia, or enuresis when coughing; ⑧ The tongue is light, or the pulse is thin. On the basis of any two items among ①, ② and ③, other plus three items among ④, ⑤, ⑥, ⑦, and ⑧ should also be met.

### 2.3 Study procedure

After a washout period of 2 weeks, participants were randomly assigned into traditional Chinese medicine group (TCMG) or control group (CG) with 1:1 to receive CM granules or the placebo of CM granules for 26 weeks. CM granules have been applied according to TCM syndromes including Bao-fei Hua-xian granule for lung qi deficiency syndrome, Jin-shui Huan-xian granule for lung-kidney qi deficiency syndrome, and Yang-qing Kang-xian granule for yin deficiency and inter heat syndrome. A follow-up visit would be managed in every 13 weeks during the treatment period.

**FIGURE 1:**
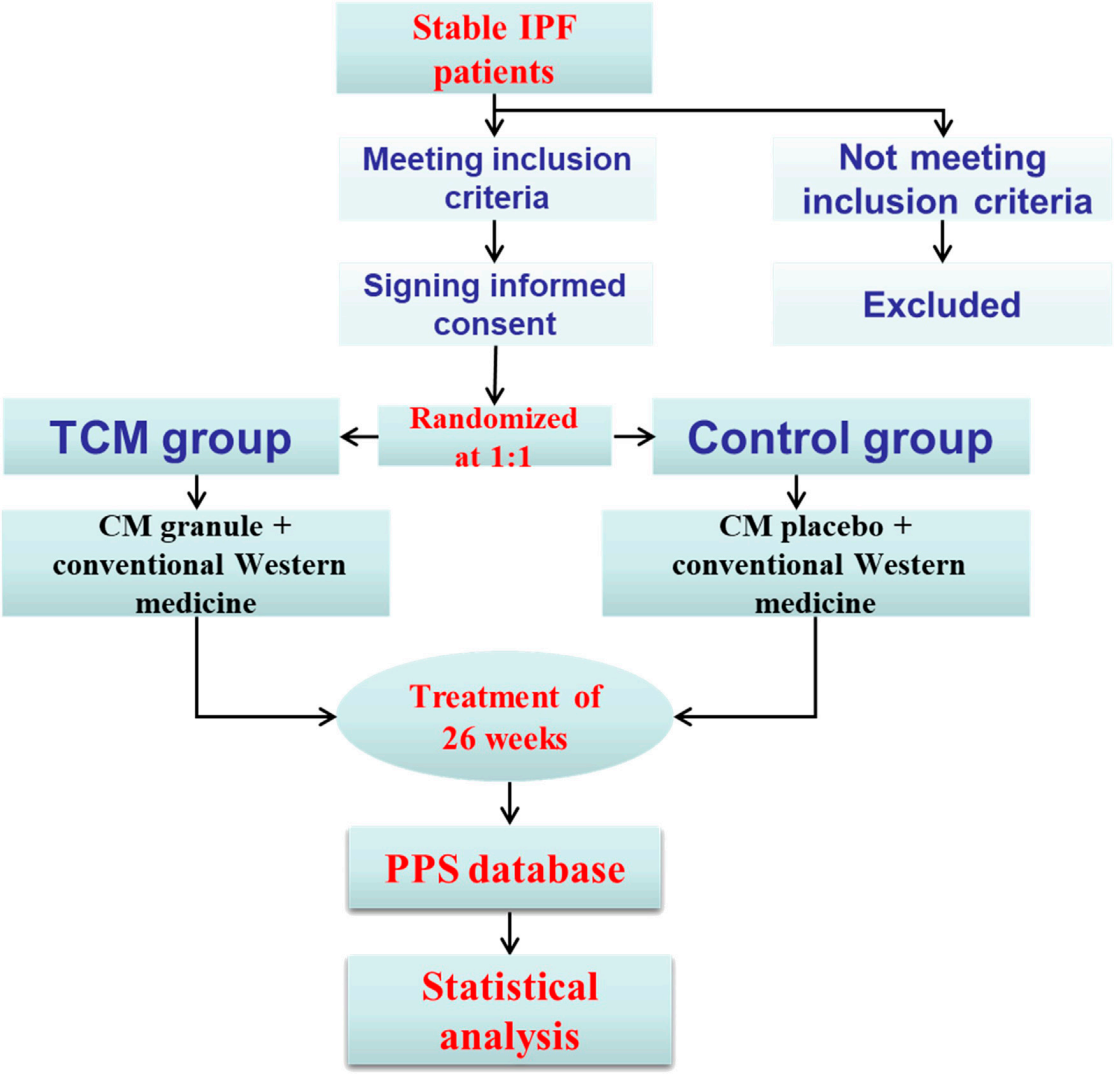
Study procedure of the trial.

Central randomization was adopted by third party organization through SAS9.2 software. Patients, investigators, data collectors and the study sponsor were all blind to the treatment assignments in the whole process of study. To minimize the missing data, patients, who discontinued the trial, would be contacted through phone or other ways as soon as possible and complete all the questionnaires that should be completed. Any adverse event, which would be reported and dealt with in 24 h, was centrally collected. Informed consent and record should be completed for no uniform appointment on the application of pifenidone and nintedanib. If necessary, the symptomatic medication, such as ambroxol hydrochloride tablets, compound methoxyphenamine capsules and budesonide formoterol inhalants, would be applied. All the enrollments and follow-up visits have been managed in a special place. The study procedure could be found in Figure 1.

### 2.4 Outcomes

The primary outcomes were pulmonary function and AEs of IPF. Forced expiratory volume in one second (FEV1) and forced vital capacity (FVC) were measured in milliliter. Carbon monoxide diffusion capacity (DLCO) was measured in a percentage of the predicted value (DLCO%), and annual numbers of AE were also calculated. The diagnosis of AEs should meet the criterion published by the American Thoracic Society (Collard et al., 2016).

The secondary outcomes included clinical symptoms, dyspnea scores, HRQoL, 6MWT and all-cause mortality. The clinical symptoms were measured by cough, expectoration, chest tightness, shortness of breath, fatigue, and cyanosis, with the scoring criteria in Table 1. Dyspnea score was assessed with modified Medical Research Council (mMRC). HRQoL was assessed by the total score of the St. George’s Respiratory Questionnaire (SGRQ), COPD assessment test (CAT)and short form 36 (SF-36) health survey questionnaire. Pulmonary function test was conducted at baseline and 26 weeks. 6MWT, mMRC, SGRQ, CAT, and SF-36 were assessed at baseline and every follow-up visit. All-cause deaths were counted separately to calculate all-cause mortality at 26 weeks.

**TABLE 1:** Scoring criteria of clinical symptoms and signs.

	0	1	2	3
Cough	No	Only in the morning	Through the day	Frequent cough through the day
Expectoration	No	10–20 ml through the day	20–30 ml through the day	30 ml and above through the day
Chest tightness	No	Once in a while	Often and post-activity aggravation	Obvious with incidence even rest
Shortness of breath	No	With heavy activity	With light activity	Even rest
Fatigue	No	Light	Moderate	Severe
Cyanosis	No	Light	Moderate	Severe

Safety was assessed by routine blood, urine and stool test, liver and kidney function, electrocardiogram and adverse events. Routine blood, urine and stool test, liver and kidney function, electrocardiogram will be evaluated pre- and post-treatment. Adverse events will be recorded and dealt with as soon as possible.

### 2.5 Statistical analysis

Estimation method of sample size for exploratory research was adopted. And a lost-rate of 20% was also taken into account. The target total sample size was 80. Both efficacy and safety analysis were managed for all the patients in the trial. SAS 9.2 statistical software was applied in data analysis. The measurement data with normal distribution were represented by mean ± standard deviation ( ‾ *x* ± s), as with non-normal distribution represented by median ± quartile (M ± Q). Paired *t*-test was applied in the comparison within group with normal distribution and homogeneous data, as Wilcoxon rank sum test used with non-normal distribution or non-homogeneous data; Variance analysis or independent sample *t*-test was applied in the comparison between groups with normal-distribution and homogeneous data, as Wilcoxon rank sum test used with non-normal distribution or non-homogeneous data. The counting data were described by frequency, number of incidence or constituent ratio, and the differences among groups was tested by contingency table chi-square test. Two-sided significance tests with an alpha value of 0.05 have been adopted. All the statistical analysis would be performed by third-party professional statisticians named Jiangsu Famous Medical Technology Co. Ltd. in Nanjing, China.

## 3 Results

### 3.1 Baseline characteristics of patients

**FIGURE 2:**
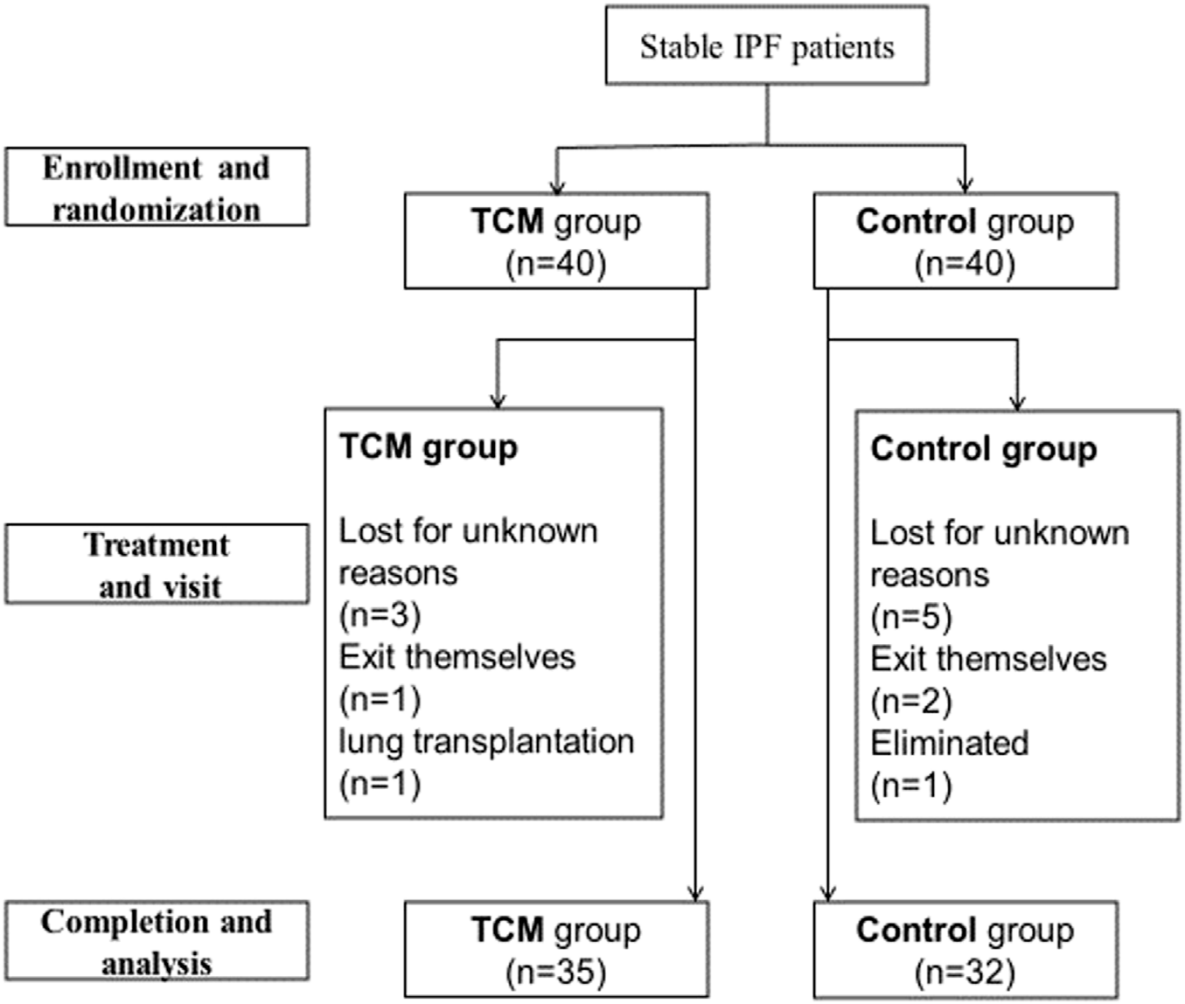
Presentation of patients’ completion in the study.

From August 2016 to June 2017, a total of 80 stable IPF patients from outpatient of the above four sub-centers were enrolled (Figure 2). Among these patients, five in TCMG and seven in CG discontinued the trial, and one in CG was eliminated because of not taking medicine. Statistical analysis was completed on data from 35 patients in CMG and 32 in CG (Figure 2). Five patients in TCMG and one in CG took pifenidone with no statistical difference. There were also no statistical differences between the two groups in sex, nation, ages, occupations, education levels, smoking histories, disease durations, pulmonary function, annual incidences of acute exacerbation, mMRC, 6MWT, and HRQoL (Table 2).

**TABLE 2:** General characteristics of patients at baseline^b^.

Characteristics	TCM group	Control group	*χ* ^*2*^ */F*	*p*
Average age—years^a^	64.54 ± 6.50	63.00 ± 8.32	−0.849	0.399
Male sex—no. (%)	21 (60.0)	22 (68.75)	0.557	0.456
Han nationality—no. (%)	34 (97.14)	31 (96.88)	0.004	0.949
Education levels				
Illiteracy/Semiliterate—no. (%)	1 (2.86)	1 (3.13)	9.596	0.143
Primary school—no. (%)	8 (22.86)	9 (28.13)		
Junior middle school—no. (%)	7 (20.00)	11 (34.38)		
Senior middle school—no. (%)	7 (20.00)	8 (25.00)		
Junior college—no. (%)	10 (28.57)	1 (3.13)		
Undergraduate—no. (%)	2 (5.71)	1 (3.13)		
Postgraduate—no. (%)	0 (0)	1 (3.13)		
Occupations				
Worker—no. (%)	2 (5.71)	2 (6.25)	2.763	0.838
Farmer—no. (%)	10 (28.57)	13 (40.63)		
Intellectual—no. (%)	1 (2.86)	1 (3.13)		
Manager—no. (%)	2 (5.71)	0 (0)		
Service—no. (%)	1 (2.86)	1 (3.13)		
Retirement—no. (%)	18 (51.43)	14 (43.75)		
Unemployed—no. (%)	1 (2.86)	1 (3.13)		
Patients with smoking history—no. (%)	18 (51.43)	17 (53.13)	0.019	0.89
Patients with co-morbidity—no. (%)	6 (17.14)	9 (28.13)	1.16	0.281
Diseases duration—months^a^	27.97 ± 27.39	24.98 ± 25.69	−0.459	0.648
Annual numbers acute exacerbation^a^	1.06 ± 1.08	1.00 ± 1.07	−0.216	0.829
Scores of mMRC^a^	1.71 ± 0.66	1.56 ± 0.71	−0.898	0.372
6MWT—m^a^	342.57 ± 81.18	374.38 ± 87.67	1.542	0.128
Symptoms and signs total scors	8.54 ± 3.00	8.41 ± 2.38	0.651	0.838
mMRC scors	1.71 ± 0.66	1.56 ± 0.71	−0.898	0.372
CAT total scors	15.06 ± 6.66	13.22 ± 5.66	1.212	0.230
SGRQ total scors	47.86 ± 15.33	45.50 ± 17.92	0.314	0.563
SF-36 total scors	104.09 ± 15.73	104.91 ± 12.40	1.397	0.815
Lung function				
FEV 1—L	1.91 ± 0.62	1.99 ± 0.54	0.565	0.574
FVC—L	2.36 ± 0.76	2.42 ± 0.83	0.312	0.756
DLCO%—%	40.26 ± 13.60	44.18 ± 15.73	0.453	0.278

^a^ Plus–minus values are means ± SD.

^b^ There were no statistical differences between the two groups in any characteristic at baseline.

### 3.2 Acute exacerbations of idiopathic pulmonary fibrosis

**FIGURE 3:**
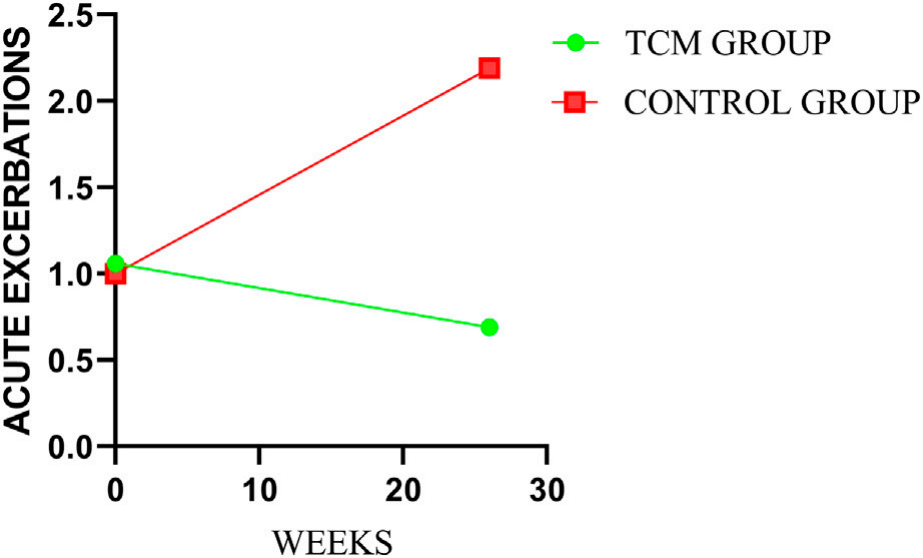
Comparison of differences between groups on AEs.

After treatment, there was statistical difference between the two groups in annual numbers of AEs (*p* = 0.0008). And the mean annual numbers were 0.69 and 2.19 times in TCMG and CG (mean difference, −1.50 times; 95%CI, −2.60 to 0.40), respectively. The mean changes from baseline in annual numbers of AEs were −0.37 and 1.19 times in TCMG and CG (mean difference, −1.56 times; 95% CI, −2.69 to −0.43). Annual number of AEs was significantly higher in CG than TCMG after treatment. Details could be got in Table 3 and Figure 3.

**TABLE 3:** Efficacy outcomes assessment after treatment.

Outcomes	TCM group		Control group		Difference (95% CI)	*p*
	No. of patients	Mean change from baseline^a^	No. of patients	Mean change from baseline^a^		
Annual numbers of AEs	35	−0.37 ± 1.96	32	1.19 ± 2.66	−1.56 (−2.69, −0.43)	0.01
Pulmonary function						
FEV1(L)-at week 26	35	−0.06 ± 0.22	32	−0.10 ± 0.20	0.05 (−0.06, 0.15)	0.38
FVC(L)-at week 26	35	−0.10 ± 0.27	32	−0.11 ± 0.24	0.01 (−0.11, 0.13)	0.86
DLCO%-at week 26	35	4.40 ± 9.89	32	−0.88 ± 8.66	5.29 (0.76, 9.81)	0.02
Clinical symptoms and signs						
Cough score						
At week 13	35	−0.14 ± 0.65	32	0.03 ± 0.54	−0.18 (−0.47, 0.12)	0.24
At week 26	35	−0.26 ± 0.78	32	0.13 ± 0.61	−0.38 (−0.73, −0.04)	0.03
Expectoration score						
At week 13	35	0.00 ± 0.91	32	0.19 ± 0.64	−0.19 (−0.58, 0.2)	0.34
At week 26	35	0.00 ± 0.94	32	0.25 ± 0.72	−0.25 (−0.66, 0.16)	0.23
Chest tightness score						
At week 13	35	−0.23 ± 0.77	32	−0.03 ± 0.54	−0.20 (−0.52, 0.13)	0.23
At week 26	35	−0.20 ± 1.11	32	−0.13 ± 0.66	−0.08 (−0.53, 0.38)	0.74
Shortness of breath score						
At week 13	35	−0.06 ± 0.64	32	−0.06 ± 0.62	0.01 (−0.30, 0.31)	0.97
At week 26	35	−0.26 ± 0.78	32	−0.03 ± 0.70	−0.23 (−0.59, 0.14)	0.22
Fatigue score						
At week 13	35	−0.26 ± 0.70	32	−0.13 ± 0.71	−0.13 (−0.48, 0.21)	0.45
At week 26	35	−0.37 ± 0.97	32	−0.16 ± 0.68	−0.22 (−0.63, 0.20)	0.30
Cyanosis score						
At week 13	35	−0.17 ± 0.51	32	−0.03 ± 0.54	−0.14 (−0.40, 0.12)	0.28
At week 26	35	−0.29 ± 0.57	32	−0.13 ± 0.55	−0.16 (−0.44, 0.12)	0.25
mMRC score						
At week 13	35	−0.31 ± 0.80	32	−0.16 ± 0.72	−0.16 (−0.53, 0.21)	0.40
At week 26	35	−0.43 ± 1.07	32	−0.22 ± 0.79	−0.21 (−0.67, 0.25)	0.37
HRQoL						
CAT total score						
At week 13	35	−1.17 ± 6.90	32	0.28 ± 5.62	−1.45 (−4.54, 1.63)	0.35
At week 26	35	−1.60 ± 7.50	32	0.19 ± 6.87	−1.79 (−5.31, 1.73)	0.31
SF-36 total score						
At week 13	35	5.91 ± 12.31	32	6.35 ± 13.36	−0.44 (−6.70, 5.82)	0.89
At week 26	35	6.62 ± 16.50	32	6.12 ± 13.91	0.50 (-6.98, 7.98)	0.89
SGRQ total score						
At week 13	35	−5.02 ± 15.44	32	−1.29 ± 11.76	−3.73 (−1078, 3.01)	0.27
At week 26	35	−5.66 ± 18.04	32	−0.86 ± 10.69	−4.80 (−12.13, 2.52)	0.20
6MWD (m)						
At week 13	35	14.11 ± 34.26	32	−8.91 ± 39.28	23.02 (5.07, 40.97)	0.01
At week 26	35	24.46 ± 63.07	32	−5.97 ± 48.18	30.43 (2.85, 58.00)	0.03
All-cause mortality	No. of patients	No. of patients with events (%)	No. of patients	No. of patients with event (%)	Hazard Ratio (95% CI)	
	35	3 (8.6)	32	7 (21.9)	0.39 (0.10, 1.52)	0.13

^a^ mean ± SD.

Data presented as between-time mean (95% CI) differences by groups.

6MWT, 6-min walk test; AE, acute exacerbation; CAT, COPD assessment test; DLco%, diffusing capacity percentage of the predicted value; FEV1, forced expiratory volume in one second percentage; FVC, forced vital capacity; HRQoL, health-related quality of life; mMRC, modified medical research council; SF-36, 36-item short-form health survey; SGRQ, St. George’s respiratory questionnaire.

### 3.3 Pulmonary function

**FIGURE 4:**
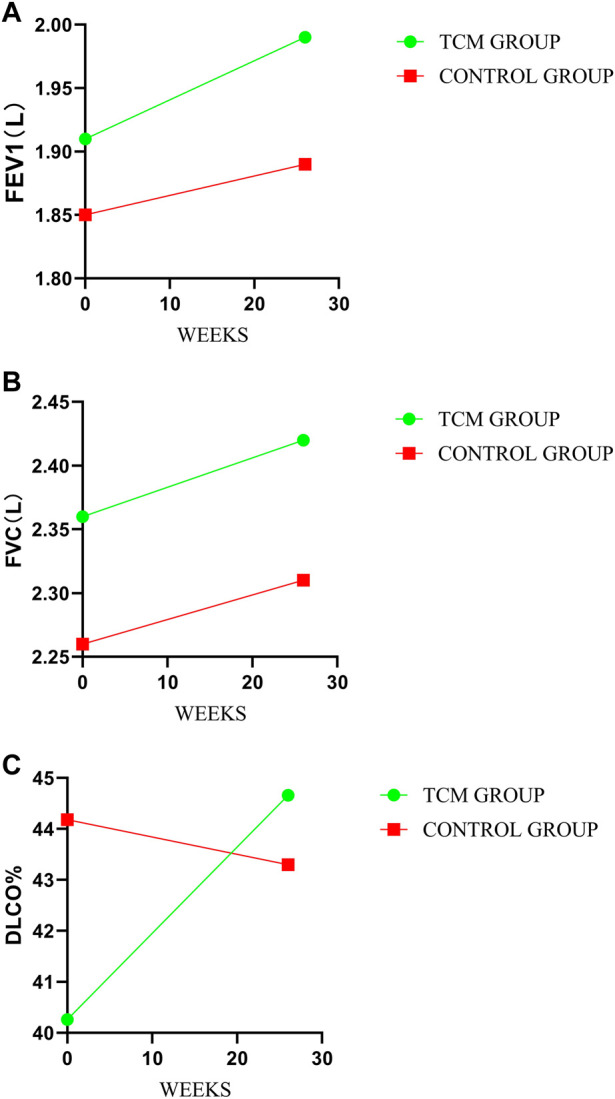
Comparison of differences between groups on pulmonary function. **(A–C)** represent the treatment change trends of FEV1, FVC, and DLco%, respectively.

There were no significant differences in FVC and FEV1 between TCMG and CG. However, a meaningful difference was shown in DLCO%. The mean changes from baseline in DLCO% were 4.40 and −0.88 (mean difference, 5.29; 95% CI, 0.76 to 9.81, *p* = 0.02) in TCMG and CG, respectively. Other results could be found in Table 3 and Figure 4.

### 3.4 Clinical symptoms and signs

**FIGURE 5:**
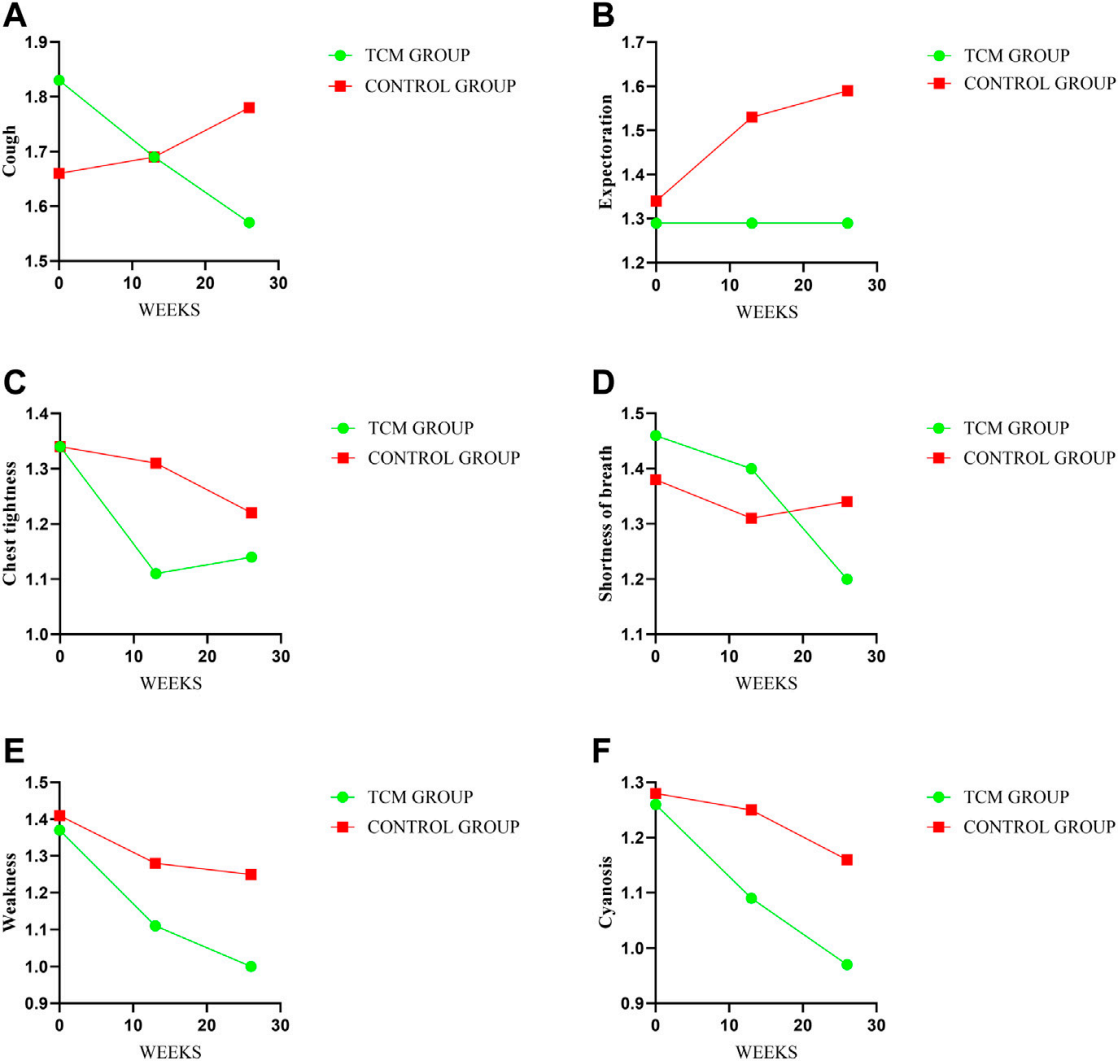
Comparison of differences between groups on symptoms and signs. Treatment change trends of cough, expectoration, chest tightness, shortness of breath, weakness and cyanosis have been shown in **(A–F)** respectively. The higher score will reflect the worse symptoms.

There were no meaningful inter-group difference in clinical symptoms and signs expect cough. The mean changes from baseline in cough score were −0.14 and 0.03 points in TCMG and CG (mean difference, −0.18 points; 95% CI, −0.47 to 0.12) at week 13 and −0.26 points and 0.13 points at week 26 (mean difference, −0.38 points; 95% CI, −0.73 to −0.04, *p* = 0.03), respectively. Other details were shown in Table 3 and Figure 5.

### 3.5 mMRC

**FIGURE 6:**
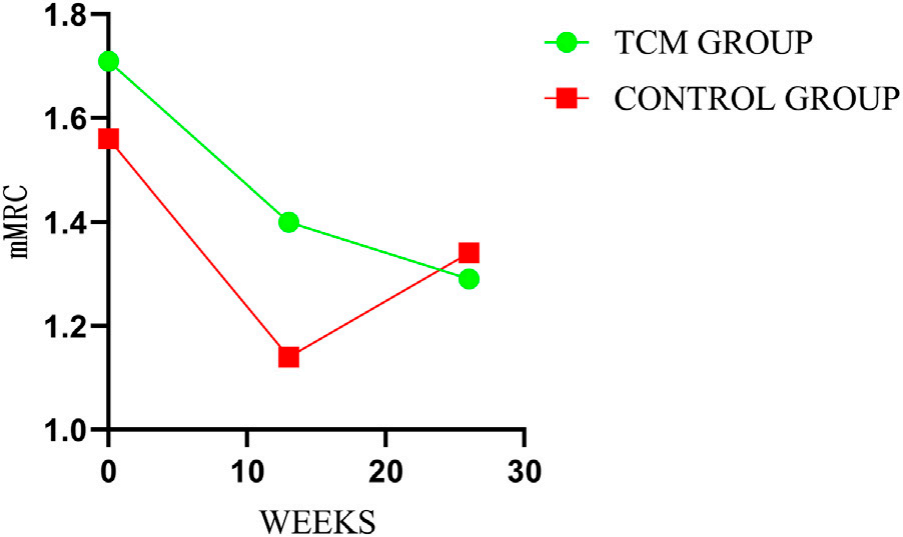
Comparison of differences between groups on mMRC.

There was no significant inter-groups difference in mMRC scores. However, there were intra-group significant differences in TCMG between three or 6 months after treatment and baseline, with no intra-group significant differences for CG. As shown in Table 3, the mean changes in mMRC score from baseline were −0.31 points in TG and −0.16 points in the CG at week 13 (mean difference = −0.16 points, 95% CI,−0.53 to 0.21; *p* = 0.40), and −0.43 points and −0.22 points at week 26 (mean difference = −0.21 points, 95% CI, −0.67 to 0.25; *p* = 0.37), respectively. Details could be found in Table 3 and Figure 6.

### 3.6 All-cause mortality

**FIGURE 7:**
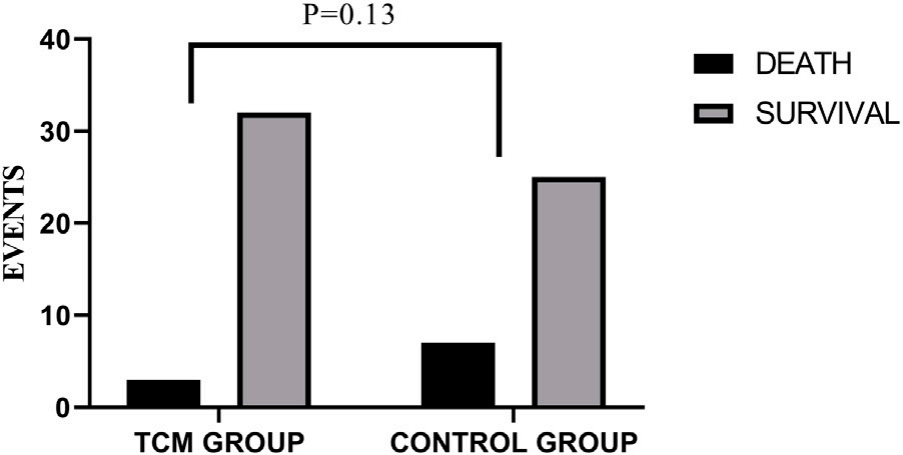
Comparison of differences between groups on mortality.

Three of the 35 patients in the TCMG died in the whole trial period, with seven of 32 patients in the CG. Harzard Ratio (HR) for CM granules in all-cause mortality is 0.39 (95% CI, 0.10–1.52) (Table 3). Please found details in Table 3 and Figure 7.

### 3.7 Health-related quality of life

**FIGURE 8:**
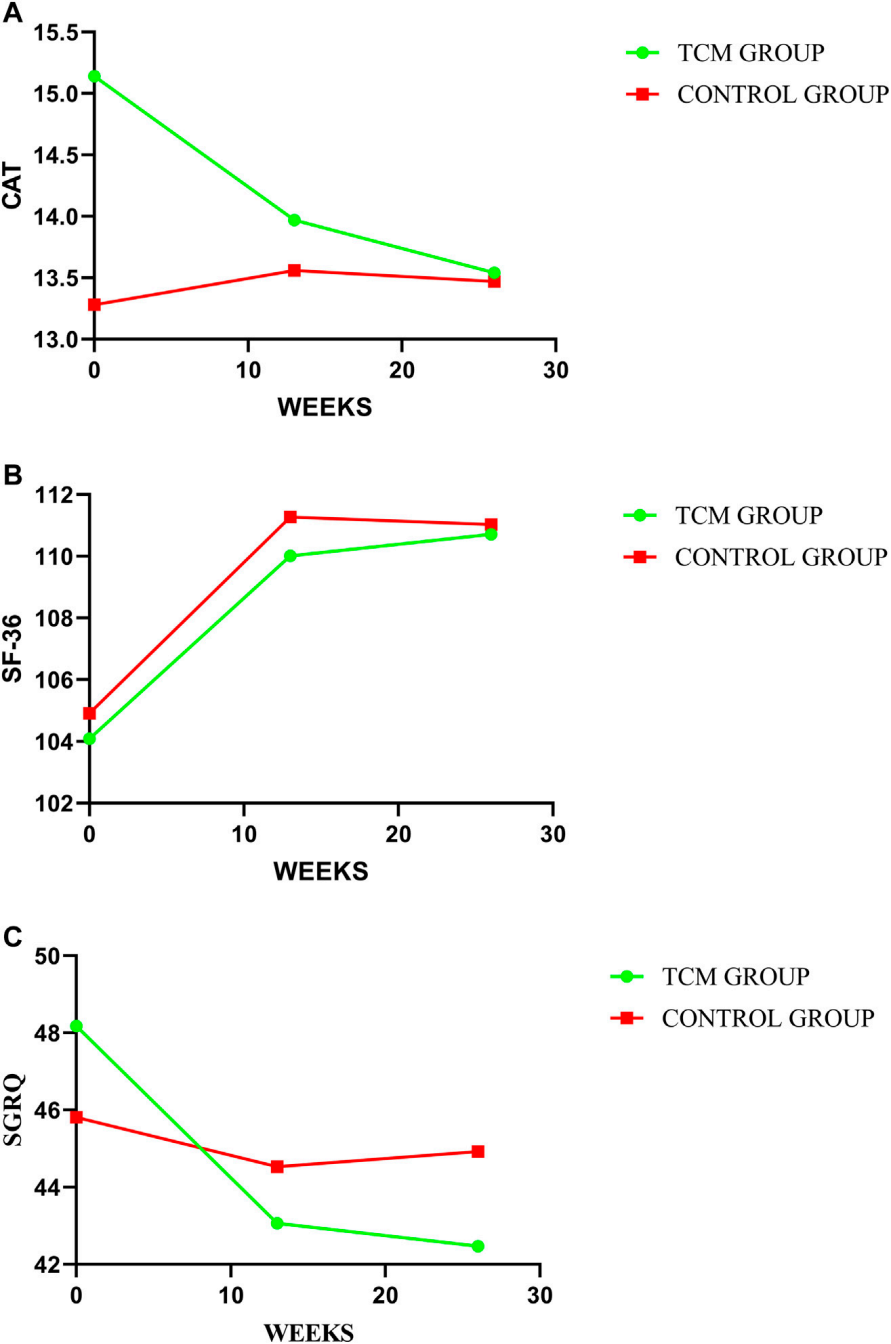
Comparison of differences between groups on HRQoL. In this study, HRQoL has been assessed by CAT, SF-36, and SGRQ, with treatment change trends shown in (A–C) respectively. A higher value indicate a better HRQoL for SF-36 with a worse HRQoL for CAT and SGRQ.

In our study, no significant between-group difference has emerged in HRQoL assessed by SGRQ, CAT, and SF-36. However, as shown in Table 3, the results indicated a trend of improvement in HRQoL in both groups, and the TCMG may be better than CG. See details in Table 3 and Figure 8.

### 3.7 6-min walk test

**FIGURE 9:**
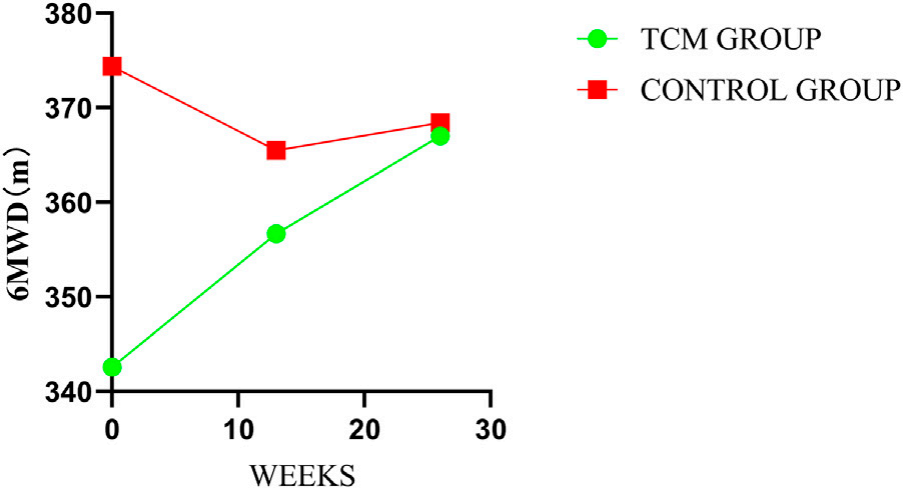
Comparison of differences between groups on 6MWD.

In the treatment period, there were significant inter-group differences in mean change at every follow-up visit and post-treatment in 6MWT. The mean changes from baseline were 14.11 m in TCMG and −8.91 m in CG (mean difference = 23.02 m; 95% CI, 5.07–40.97, *p* = 0.01) at week 13 and 24.46 m and −5.97 m in respectively (mean difference, 30.43 m; 95% CI, 2.85–58.00, *p* = 0.03) at week 26. Details have been presented in Table 3 and Figure 9.

### 3.8 Adverse events

In the whole trial, very few patients suffered from transient gastric discomfort, and no other significant adverse events were observed. None of adverse events were assessed to be related to the study drug.

## 4 Discussion

To our knowledge, this is the first randomized, controlled trial showing efficacy and safety of CM granules in IPF. Throughout the trail, only a few patients were prescribed with anti-fibrosis medicines. The efficacy and advantages of TCM in the treatment of IPF were initially highlighted. The results indicated that TCM granules could reduce incidences of AEs and risk of death, slow down the decline of pulmonary function, and improve HRQoL and exercise capacity for IPF to some extent. No obvious adverse reactions have been observed. However, the clinical symptoms and signs have also been improved to some extent with no statistical significance expect for cough.

AEs, which could usually induce worsen HRQoL, disease-progression and even death (Collard et al., 2016; Kakugawa et al., 2016; Qiu et al., 2018), has been a primary outcome to assess efficacy of CM granules in the study. We could find that incidences of AEIPF in CG increased with the course of disease, as was no obvious trend in TG. CM granules can reduce incidences of AEs.

Pulmonary function, which is the most susceptible physiological function for IPF patients, is also the common efficacy indicator. So, it has also been another primary outcome. DLCO or DLCO% may be the most sensitive and be consistent with prognosis (Behr et al., 2009). FVC can also indicate the disease-progression and prognosis (Salisbury et al., 2016) and also be applied in other researches (King et al., 2014; Richeldi et al., 2014). Our study find that FVC and FEV1 decrease in both groups, with more pronounced in CG than that in TCMG. And DLCO% rose in TCMG as decreased in CG. CM granules may delay the deterioration of pulmonary function for IPF patients.

IPF is a critical disease with a poor prognosis and high-mortality. So some scholars insist that it could be applied as main outcome in the clinical study for IPF (Raghu et al., 2012; Collard et al., 2014). However, other scholars insist the converse opinion (Wells et al., 2012). They deem that a longer treatment period would be needed if death was applied as outcome. This may be the possible reason why there is no inter-group difference in all-cause mortality in this study.

IPF patients often undergo with poor HRQoL (Hopkins et al., 2016). So, improving HRQoL is also one of the goals for IPF. In our study, HRQoL, which was assessed by SF-36, CAT and SGRQ, has also been adopted as outcomes. There are no inter-group differences in SF-36, CAT and SGRQ, with some intra-group differences and better improvement trends in TCMG. The results indicated that CM granules may have improvement in HRQoL for IPF.

For IPF, some clinical symptoms and signs including cough, expectoration, chest tightness and so on, often occur in the process of disease. According to TCM theory, CM granules is based on clinical symptoms and signs. The improvement in clinical symptoms and signs should be the first step. However, the results showed that CM granules has a good tendency in improving symptoms and signs with no statistical difference. This may also indicate that clinical symptoms and signs are difficulty to improve for IPF.

Exercise capacity, which is assessed by 6MWT in this study, will decrease with disease progression in IPF. It is also an important indicator of disease severity and HRQoL in IPF (Bahmer et al., 2017). And because of its simple operation and good repeatability (du Bois et al., 2014; Holland et al., 2018), 6MWT has been applied to assess exercise capacity and clinical efficacy (Holland et al., 2014; Baddini-Martinez, 2018). Our results indicate that CM granules could improve 6MWT in IPF. The result may also indicate that CM granules could improve exercise capacity, HRQoL and disease severity.

In addition, characteristic changes in HRCT are the critical and objective efficacy and prognosis references for IPF evaluation. However, at the beginning of this study, the efficacy evaluation value of HRCT has not been highlighted, and not been adopted by the contemporary international studies. Therefore, the deficiency remains in this study, which will be improved in the future researches.

Although more and more relevant treatment researches have been conducted or being conducted in IPF, only pifenidone and nintedanib obtained evidence-based efficacy and were recommended in the guidelines up to now (King et al., 2014; Richeldi et al., 2014; Raghu et al., 2015; Behr, 2016; Cottin et al., 2017; Behr et al., 2018; Homma et al., 2018; Jo et al., 2018; Lee et al., 2019; Raghu et al., 2022). Drug-related side effects and their high prices have also caused limitations for clinical application. Other effective treatments are still needed to be developed. The pathogenesis of IPF has not been completely clear, so the drug development has also been restricted. The advantages of CM granules emerged. According to TCM theory, names of diseases, TCM pathogenesis and syndromes should be determined according to clinical symptoms and signs first. And then, the treatment would be completed. The process is called TCM syndrome differentiation. So, the pathogenesis in Western medicine is not necessary. The application of CM granules is more convenient and worth to promote. In addition, compared with the anti-fibrosis drugs being used, pifenidone and nintedanib, CM may also have the health economics advantage with cheap price. In our study, we could also find that, the adverse reactions of CM were few and well tolerated. More IPF patients will also have another treatment option and benefited in the absence or intolerance of anti-fibrosis treatment.

As we known, blood stasis and phlegm turbidity are also important pathological factors. According to the standards issued in 2012 by the Professional Committee of Pulmonary Diseases of China Association of Chinese Medicine, the syndromes for IPF include three types, which have seven syndromes, namely, deficiency syndrome (lung qi deficiency, yin deficiency and internal heat, lung and kidney qi deficiency, lung and kidney qi yin deficiency), excess syndrome (phlegm heat obstructing the lung, phlegm turbidity obstructing the lung), and concurrent syndrome (blood stasis). However, because of small sample, not all the syndromes could be taken into account. So, this study only selected the main TCM syndromes, including patterns of lung qi deficiency, lung-kidney qi deficiency, and yin deficiency inner heat, for observation on the basis of previous research, and there are also relevant single Chinese medicine for reducing phlegm and blood circulation given in the compound CM granules. Other syndromes will be considered in the future researches.

The sample size is also too small for this study as a multicenter research. However, IPF is a rare disease, and it is difficult to enroll patients to participate the study in clinical practice. Then, this is an exploratory trial without sufficient preliminary data support. The sample size cannot be estimated exactly. So, we estimated sample size referring to sample size estimation method for exploratory clinical trial and to provide references for further confirmatory study.

Although some achievements have been obtained, there are still some limitations in the study. First, as an exploratory study with a deficiency of foundation, this trial does not have an ample sample size, which may also be the possible reason for that there was no statistical significance in some results. Second, although CM granules brings an advantage for TCM with no need for clear and complex pathogenesis of Western medicine, its shortcomings has also appeared. TCM syndromes often depend on the subjective judgement by researchers, with a deficiency of objective indicators. So, further confirmative researches with appropriate sample size and core TCM pathogenesis need to be carried out in the future. Developing objective indicators combining of IPF and TCM syndromes will also be another direction as well as mechanism of TCM in IPF.

## Data Availability

The datasets presented in this article are not readily available because the follow-up research is in progress. Data sharing will be available at the end of all studies. Requests to access the datasets should be directed to JL, li_js8@163.com.
